# Industrial green transformation efficiency and its driving factors in resource-based cities: the case of the Yellow River Basin

**DOI:** 10.1038/s41598-026-57086-0

**Published:** 2026-06-15

**Authors:** Yang Yuchun, Liang Jiayao

**Affiliations:** School of Economics, Northwest Normal University, Lanzhou City, Gansu Province China

**Keywords:** Resource-based cities, Industrial green transformation efficiency, Driving factors, The Yellow River Basin, Environmental sciences, Environmental social sciences, Environmental studies

## Abstract

Enhancing industrial green transformation efficiency (IGTE) of resource-based cities in the Yellow River Basin is critical for upgrading industries, controlling carbon emission, and fostering sustainable high-quality development. This study evaluates the IGTE of 40 resource-based cities within the basin from 2010 to 2021 using the Super-SBM model, and analyzes its spatiotemporal evolution. Furthermore, the Tobit model is employed to identify the driving factors of IGTE. The results indicate that: (1) During the study period, the IGTE of resource-based cities shows a fluctuating upward trajectory. From a spatial perspective, cities located in the lower reaches perform better than those in the middle and upper reaches. Regarding development stages, growing cities achieve the highest level of IGTE, followed by regenerative ones, whereas mature and declining cities exhibit comparatively lower efficiency. (2) At the basin-wide level, both economic development and environmental regulations exert significant positive effects on IGTE. (3) For mature cities, industrial structure upgrading and industrial agglomeration contribute positively to IGTE improvement. In contrast, technological advancement and industrial structure show no significant promoting effect in declining and regenerative cities. Based on these results, this paper proposes targeted policy recommendations to facilitate a balance between economic growth and environmental government in ecologically vulnerable regions like the Yellow River Basin.

**Supplementary Information:**

The online version contains supplementary material available at 10.1038/s41598-026-57086-0.

## Introduction

The Yellow River Basin represents a critical ecological barrier and a major base for energy and industrial development in China^1^. Endowed with abundant reserves of coal, petroleum, natural gas, and non-ferrous metals, the region hosts a significant number of resource-based cities—those dependent on the extraction and processing of natural resources—which account for over 50% of all cities in the basin. Although resource exploitation has substantially propelled economic growth in these areas, the region’s inherently fragile ecosystem and its resource-intensive development model have imposed considerable ecological pressures and raised sustainability challenges for local industries. Industrial production is the biggest contributor to energy consumption and contamination of the environment. Against this backdrop, identifying pathways for promoting industrial green transformation of resource-based cities in the basin has become an urgent priority^2,3^.

The 14th Five-Year Plan for Industrial Green Development emphasizes the need to coordinate economic growth with the green transition, with a particular focus on improving energy efficiency and fostering low-carbon development capabilities in the industrial sector. Furthermore, the report of the 20th National Congress of the Communist Party of China highlighted the importance of advancing the clean, low-carbon, and efficient use of energy resources, accelerating the development of green technologies in the industry, and facilitating the shift toward cleaner and low-carbon industrial processes. Industrial green transformation (IGT) is a sustainable industrialization model, guided by resource-intensive utilization and environmental friendliness and propelled by innovation, which seeks to enhance the entire production chain for combined economic-ecological gains^4^. Resource-based cities in the Yellow River Basin generally face practical challenges such as high resource dependence, strong ecological constraints, and a heavy industrial structure. Measuring industrial green transformation efficiency and its determinants in cities at various regional levels and development stages offers empirical evidence to support local governments in designing differentiated and actionable green transformation policies. It helps resource-based cities break away from path dependence, improve resource utilization efficiency, and reduce pollution emissions, thereby maintaining the bottom line of ecological security while ensuring stable industrial growth. Such efforts promote the coordinated advancement of ecological preservation and high-standard economic growth within the river basin, and can also serve as a practical reference for similar resource-based cities and key river basins nationwide in their green transformation endeavors. In the context of worsening global climate change, enhancing industrial green transformation efficiency has emerged as a key metric for assessing the industrial system’s capacity to cope with external environmental risks^5^. Enhancing IGTE is central to balancing environmental conservation and regional development. Thus, it is imperative to measure IGTE, uncover its spatiotemporal dynamics, and pinpoint improvement pathways. This research is essential for overcoming resource dependency and environmental constraints, ultimately guiding these cities toward high-quality green development in the basin.

### Literature review

Green transformation refers to a shift in development mode toward sustainability, guided by the construction of an ecological civilization, grounded in a circular economy, and supported by green governance. It aims to achieve resource conservation, environmental friendliness, ecological balance, and harmonious development among people, nature and society^6^. Industrial transformation is a fundamental requirement for following the new path of industrialization with Chinese characteristics. This new industrialization path is characterized by five distinct features: “high technological content, good economic returns, low resource consumption, minimal environmental pollution, and full utilization of human resources^7^.” These features also represent the goals of industrial transformation and upgrading. As a key component of industrial transformation and upgrading, industrial green transformation refers to a process in which industrial development is oriented toward resource-intensive utilization and environmental friendliness, driven by green innovation^8^. It involves continuously improving energy efficiency throughout the entire production process, gradually reducing pollutant emissions, and steadily enhancing sustainable development capabilities, thereby achieving a mutually beneficial result for the economy and the environment. Its ultimate goal is to decouple industrial growth from increased resource and environmental inputs, namely industrial development don’t relyon additional resource and environmental consumption.

Since the debut of the green development theory, considerable research has been conducted on the conceptual definition, measuring approaches, and driving components of the IGTE. Some scholars defined IGTE as a dynamic process in which industrial development shifts toward resource-intensive practices while lowering emission levels, minimizing negative environmental effects and enhancing resilience^9,10^. Yang and Xu^11^ further characterized it as a complete and dynamic process including the transformation of the entire financial value system with a concentration on resource conservation and sustainable development.

In terms of measurement, scholars commonly employed methods such as the Slacks-Based Measure (SBM), Epsilon-Based Measure (EBM) and Directional Distance Function (DDF) to assess IGTE. For instance, Qiu et al.^12^ combined the SBM model with a regional regression-adjustment model to examine the spatiotemporal patterns and drivers of IGTE in the Huaihai Economic Zone from 2001 to 2020. Ren et al.^13^ developed an IGTE index by integrating the SBM-Undesirable model and the Malmquist–Luenberger index, evaluating efficiency from the perspectives of developmental trends and transformation levels. Fu et al.^14^ employed a CSR approach to quantify the improvement in environmental efficiency in metropolitan cities across six provinces in China. Wang and Zhao^15^ calculated the IGTE and looked at its growth and regional dispersion using DDF method and Globle Malmquist–Luenberger index, which included urbanization variables. Yusufu and Lu^16^integrated the TOPSIS method with gray relational analysis to evaluate IGTE in cities along the Yangtze River Economic Belt and explore its spatiotemporal evolution.

However, previous research has largely concentrated on national-level analyzes or specific regions such as the Yangtze River Economic Belt^17^, the Yangtze River Delta urban agglomeration^18^, the Beijing-Tianjin-Hebei region^19^ and Northeast China^20^. Regarding influencing factors and pathways for IGTE, several studies indicated that during the revitalization period, industrial agglomeration hindered green development efficiency^21^. There is a U-shaped relationship between industrial agglomeration and green development, environmental regulations show a U-form correlation with China’s industrial green growth^22^. Some scholars have found that R&D intensity is a factor that exerts a highly significant and positive influence on GTFP and TFP growth in study period^23^. Existing studies have shown that climate change can significantly affect productivity with clear spatial heterogeneity across cities in China^24,25^. According to Jing et al.^26^, there is a considerable inverted N-shaped nonlinear relationship between industrial green total factor productivity and environmental restrictions, and the integration of demand-based, funding-based and cost-based regulations regarding the environment can support environmentally conscious and sustainable industrial transformation. Liu et al.^27^ found that foreign direct investment had an inverted U-shaped impact on IGTE, with significant spatial spillover effects. Wu and Zhang^28^ suggested that foreign investment, advanced industrial structure and urbanization significantly enhance regional manufacturing green development efficiency. He and Yang^29^ found that technical efficiency and technological progress exert heterogeneous effects across upstream, midstream, and downstream river basins, with technological progress being particularly conducive to high-level green total factor productivity. Zhai and An^30^ underlined the beneficial effect of labor in driving the green reformation of the industrial industry. New pathways for greening industry have also emerged in recent years thanks to the evolution of the digital economy and artificial intelligence. On the one hand, emerging technologies such as industrial internet, cloud computing, big data, and digital inclusive finance, serving as important engines of economic development in the new era, can optimize the efficiency of industrial resource allocation at both the intensive and extensive margins, thereby promoting the deep integration of the real industrial economy with green and low-carbon development^31^. On the other hand, innovation driven by artificial intelligence contributes to improving green total factor productivity by enhancing energy efficiency, human capital levels, and green innovation capabilities^32^. Moreover, the evolution of technology collaboration networks for climate change mitigation has been shown to significantly enhance green innovation spillovers and industrial green transformation efficiency^33^.

Resource-based cities are those that have developed through large-scale resource exploitation driven by industrialization’s substantial demand for resources^34^. Industry serves as the leading sector in the development of these cities, following the patterns of resource-based industrial growth, which is characterized by extensive development modes featuring high input, high levels of emissions, pollution, and energy using. This makes it difficult to form an intrinsic driver for economic growth, rendering such development unsustainable. Regarding resource-based cities as a specific subject of transformation, existing research has explored the issue from multiple dimensions: first, using difference-in-differences models to confirm that sustainable development policies for resource-based cities can achieve the combined benefits of cutting pollution and reducing carbon emissions by stimulating green technology innovation, optimizing industrial structure, and reducing energy consumption intensity^35^. Second, by measuring carbon emissions and high-quality development levels in resource-based cities to reveal their spatiotemporal evolution characteristics^36^. Third, systematically reviewing research progress on the transformation of China’s resource-based cities to provide directions for improving green transformation efficiency^37^.

While academic research on IGTE is numerous, it still has limits. First, existing studies focus more on the Yangtze River Basin and economically developed regions, with relatively insufficient research on the Yellow River Basin. In contrast, analyses of resource-based cities in the Yellow River Basin remain relatively limited. These cities serve as critical barriers for national ecological security and possess distinctive resource endowments, measuring the IGTE in particularly vulnerable area. Second, most existing research examines the Yellow River Basin as an aggregate, lacking sub-regional analyses that differentiate both geographical space and the varying development stages of its resource-based cities. Third, given that resource-based cities within the basin differ in geographical location and development stage, they consequently face diverse challenges and needs. On the basis of revealing the spatiotemporal differentiation patterns of IGTE, and grounded in the distinct ecological functions of the upstream, midstream and downstream sections of the Yellow River Basin, as well as the specific transformation needs of resource-based cities at different development stages, this paper proposes more targeted and location-specific policy recommendations for enhancing IGTE. The aim is to provide a reference for implementing differentiated policies and precisely advancing ecological preservation and high-standard development across the Yellow River Basin. In summary, the main contributions of this study are as follows: First, it focuses on resource-based cities in the Yellow River Basin and systematically analyzes the key factors influencing IGTE, thereby addressing the gap in existing research regarding the study object. Second, it reveals the heterogeneous characteristics of IGTE from the dual dimensions of geographical space and development stage, compensating for the lack of such a perspective in prior studies. Third, based on the heterogeneity analysis, it proposes location-specific and differentiated policy recommendations, offering targeted suggestions for promoting the industrial green transition of resource-based cities within the Yellow River Basin. Therefore, this study holds significant theoretical value and practical relevance for advancing industrial green transformation and high-quality development in these cities.

### Research design

#### Study area

**Fig. 1:**
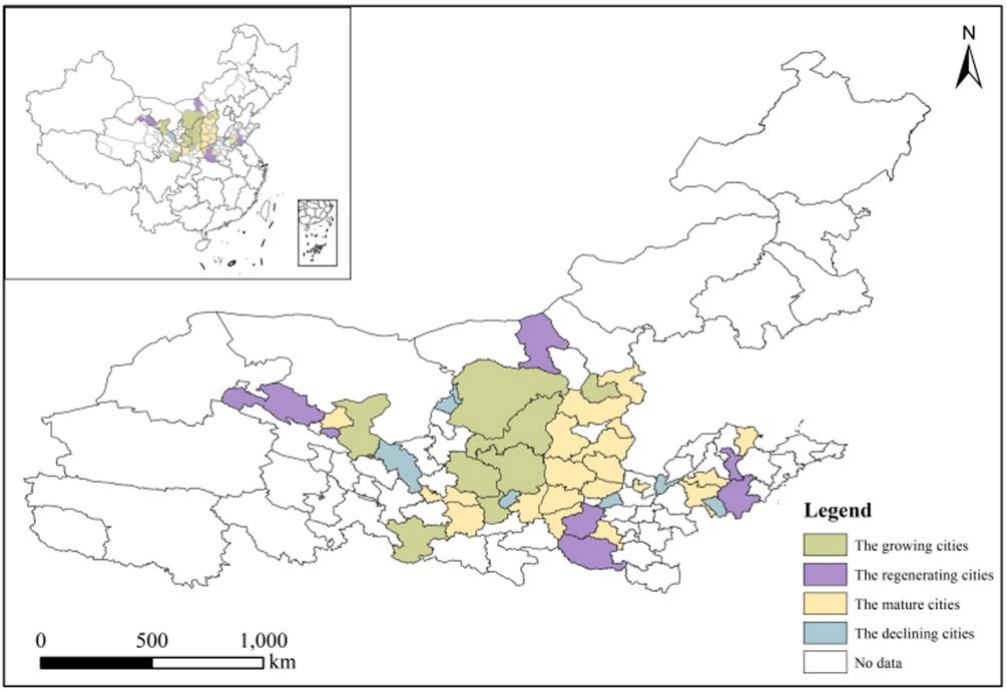
Schematic diagram of spatial distribution of research objects.

This study focuses on resource-based cities within the Yellow River Basin of China, which is conventionally defined as the area drained by the Yellow River and its tributaries. The basin spans nine provincial-level administrative regions in China: Qinghai, Sichuan, Gansu, Ningxia, Inner Mongolia, Shaanxi, Shanxi, Henan and Shandong. However, Sichuan province was excluded from the final sample due to data unavailability, given that the Yellow River flows only through a limited portion of the region, specifically, parts of Aba and Garzê Tibetan Autonomous Prefectures. In accordance with the National Sustainable Development Plan for Resource-Based Cities (2013–2020), 40 resource-based cities located within the basin were selected as the research sample. Based on their developmental life cycle, these cities were classified into four categories: growing, mature, declining and regenerating. The growing cities comprise 8 cities in total: Shuozhou City, Ordos City, Yan’an City, Xianyang City, Yulin City, Wuwei City, Qingyang City and Longnan City. The mature cities include 19 cities: Datong City, Yangquan City, Changzhi City, Jincheng City, Xinzhou City, Jinzhong City, Linfen City, Yuncheng City, Luliang City, Dongying City, Jining City, Tai’an City, Sanmenxia City, Hebi City, Pingdingshan City, Weinan City, Baoji City, Jinchang City and Pingliang City. The declining cities consist of 7 cities: Wuhai City, Zaozhuang City, Jiaozuo City, Puyang City, Tongchuan City, Baiyin City, Shizuishan City. Finally, the regenerating cities total 6 cities: Baotou City, Zibo City, Linyi City, Luoyang City, Nanyang City and Zhangye City (Fig. 1).

### Research methodology

#### Super-SBM model

The DEA model is employed to measure efficiency in multi-input, multi-output scenarios and is widely used in assessing IGTE levels. Addressing the limitation of traditional DEA models in effectively handling output slack, Tone^38^ further proposed the SBM model in 2001. This model enhances the precision of efficiency evaluation by incorporating slack variables. However, since the SBM model assesses efficiency based on a fixed frontier where efficiency typically does not exceed 1, Tone subsequently developed the Super-SBM model. This model resolves the issue of non-comparability among decision-making units (DMUs) and permits efficiency values exceeding 1. This study adopts the Super-SBM model with undesirable outputs, primarily based on the following considerations: industrial green transformation requires controlling undesirable outputs such as industrial wastewater, sulfur dioxide, and dust emissions while enhancing desirable outputs like industrial added value. The Super-SBM model can directly incorporate undesirable outputs, more realistically reflecting the “pollution and carbon reduction” essence of industrial green transformation^39^. Additionally, the model allows efficiency values to exceed 1, effectively distinguishing among efficient DMUs, providing continuous dependent variables for subsequent efficiency ranking analyses and Tobit regression, thereby reducing parameter estimation bias. In model construction, assuming a DMU comprises m input factors, n desired outputs, and q undesired outputs, the linear programming problem can be expressed as:


1


In Eq. (2), $\:{\theta\:}^{*}$represents the efficiency value of the decision unit. $\:\stackrel{-}{x}$,$\:\stackrel{-}{y}$,$\:\stackrel{-}{b}$ corresponds to the slack variables for input variables, anticipated outputs, and unanticipated outputs, respectively.


2


#### Tobit model

The Tobit model is a regression model designed to handle cases where the variable that depends is shortened or limited. This model employs maximum likelihood estimation for parameter estimation, effectively avoiding issues of inconsistent and biased parameter estimates caused by constrained variables. When using the industrial green transformation efficiency score as the dependent variable in a regression model, the issue of data truncation arises when efficiency scores are less than or equal to 0 or greater than 1^40^. In such cases, the estimates from ordinary least squares (OLS) regression are biased and inconsistent. To avoid the biases associated with OLS estimation, a limited dependent variable model is typically employed. Since the industrial green transformation efficiency scores of the 40 resource-based cities in the Yellow River Basin from 2010 to 2021 used in this study constitute panel data, we adopt the panel Tobit model. Its formula is as follows:

In the equation: $\:{Y}_{it}$ is the dependent variable; $\:{x}_{it}$ is the independent variable; $\:{\beta\:}_{0}$ is the constant term; $\:{\beta\:}_{t}\:$is the estimated coefficient vector; $\:{\epsilon\:}_{it}$ is the random error disturbance term, and $\:{\epsilon\:}_{it}$~(0,$\:{\sigma\:}^{2})$.


3


### Indicators and data

#### Indicator selection for the super-SBM model

Based on existing relevant research^41^, this study selects input indicators comprising three major factors: capital, labor, and resources^42^. Both expected and unexpected production are examples of output indicators^29^. The input- output indicator framework for IGTE in cities that rely on resources along the Yellow River Basin is constructed as shown in Table 1.

**Table 1:** Efficiency of industrial green transformation measurement index system.

Indicator index	classification	Index composition
Input indicators	Labor input	Employees in industry
	Capital investment	Total industrial fixed assets
	Energy input	Industrial water and electricity consumption
Output indicator	Expected output	Industrial added value
	Output indicator	Industrial wastewater discharge, industrial sulfur dioxide emissions, and industrial smoke (dust) emissions

#### Selection of tobit model indicators and mechanism of action

Based on existing research^43,44^ and considering the actual conditions of the Yellow River Basin, this study selects economic development level, technological level, environmental regulation, industrial structure, trade openness, and industrial agglomeration as explanatory variables. Descriptive statistics are presented in Table 2.

(1) Economic Development Level (Lnpgdp): The Environmental Kuznets Curve (EKC) theory states that widespread depletion of natural resources and increased environmental contamination usually coincide with the early phases of economic development. However, as economic development advances and industrial structure under goes optimization and upgrading, resource consumption and pollution emissions will peak before beginning to decline^45^. From the demand side, a higher level of economic development increases residents’ demand for environmental quality, thereby prompting governments and enterprises to adopt green technologies. From the supply side, the high-income stage is usually accompanied by capital deepening, which provides financial support for the renewal of clean production equipment. Higher development levels encourage industrial enterprises to prioritize green development models. This study employs logarithmically converted per capita GDP as an indicator of the level of economic development^46^.

(2) Technological Level (Lntec): The impact of technological progress on IGTE is characterized by a duality. On one hand, technological progress promotes IGTE by enhancing resource utilization efficiency and facilitating clean production, thereby reducing energy consumption and pollution emissions per unit of output. On the other hand, by lowering production costs, technological progress may induce firms to expand their production scale, leading to a scale expansion—a phenomenon particularly pronounced in the fields of resource extraction and primary processing. This expansion triggers an energy rebound, wherein the total increase in energy consumption and pollution emissions, resulting from higher aggregate output, partially offsets the environmental benefits gained from technological advancement. Consequently, when the latter dominates, technological progress may exert a negative influence on industrial green transformation efficiency. Following to Liu and Shen^47^, this analysis picks the logarithmic equation of the applications for green patents plus one for the technological level.

(3) Environmental Regulation (Er): IGTE incorporates resource and environmental constraints into traditional economic efficiency assessment frameworks. The smaller the non-desired output, the more successful the green revolution is in the sector. The degree of effectiveness of industrial sustainability increases as the volume of non-desired output decreases. Amidst tightening environmental policies^48^, industrial enterprises allocate more capital and manpower to pollution prevention and ecological conservation. This helps reduce industrial wastewater and exhaust emissions^49,50^, consequently increasing the IGTE. Following Dai Xiang et al.^51^, this investigation investigates the degree of regulation of the environment using the percentage of investment in pollution management to GDP.

(4) Industrial Structure (Is): Rationalizing industrial structure involves optimizing industrial layout to promote inter-sectoral synergy and enhance resource utilization efficiency. The tertiary sector features low resource consumption and minimal pollutant emissions, whereas the secondary sector—predominantly industrial—exhibits high resource consumption, high emissions, and high pollution. Its substantial resource usage and pollution output significantly inhibit IGTE. Existing research indicates that rationalizing industrial structure can significantly enhance the efficiency of IGTE by optimizing energy consumption patterns, the spread of clean technologies, and strengthening the effectiveness of environmental regulations^52,53^. This study analyzes the split of primary industry output to additional industry earnings for assessing the structure of the industrial sector.

(5) Trade Openness (Lntrade): Trade openness exhibits a “double-edged sword” effect. Trade openness facilitates foreign investment, introducing advanced technologies and management expertise. And it enables domestic enterprises to improve their green production capacity through the “technology spillover effect.” Also, according to the “pollution haven hypothesis,” developed countries or regions may relocate pollution-intensive industries to host countries, driving economic growth while exacerbating local environmental pollution. Given the relatively lagging economic development and the weak environmental awareness of industrial enterprises introduced by foreign-funded companies, increased trade openness may inhibit IGTE. Following Xiang Xiandi and Liu Tiantian^54^, this study uses each city’s import-export trade volume to represent trade openness, applying a logarithmic transformation to the data.

(6) Industrial Agglomeration (Lnind): Industrial agglomeration serves as a key indicator for measuring regional industrial development levels. Moderate industrial agglomeration can effectively reduce average production costs for enterprises, improve resource allocation and usage efficiency, and use economies of scale to drastically reduce resource consumption and pollutant intensity per unit of production. Through the “scale effect,” it can share the high fixed costs of pollution control facilities, making specialized environmental services possible. The economies of scale effect can also significantly reduce the intensity of resource use and pollutant discharge per unit of output, thereby positively impacting industrial green transformation efficiency. In addition, industrial agglomeration contributes to the centralized deployment of climate-resilient infrastructure, reducing adaptation costs for individual enterprises in responding to extreme climate risks^55^. As digital transformation advances, industrial enterprises within agglomerated areas can leverage industrial internet platforms to achieve synergistic optimization of energy management and pollution control, further enhancing the dynamic driving effect of industrial agglomeration on green transformation. Therefore, industrial agglomeration may be positively correlated with the efficiency of IGTE. This paper uses the number of industrial firms exceeding a designated size to reflect industrial agglomeration and applies a logarithmic transformation to this variable.

**Table 2:** Descriptive statistics of variables.

Variable	Number	Mean	Standard Deviation	Minimum	Maximum
IGTE	480	1.157	0.807	0.322	6.434
Lnpgdp	480	10.695	0.583	8.669	12.410
Lntec	480	3.892	1.380	0	6.930
Er	480	0.011	0.005	0.002	0.026
Is	480	0.856	0.491	0.194	3.524
Lntrade	480	12.614	1.810	7.800	16.817
Lnind	480	6.096	1.005	3.611	8.384

#### Data sources

The data in this paper primarily originate from the China Industry Statistical Yearbook, China City Statistical Yearbook, and Water Resources Bulletin from 2010 to 2022. To eliminate price fluctuations, per capita GDP and industrial output value were deflated to constant 2010 prices. Missing values for specific years were imputed using linear interpolation.

## Empirical analysis

### Analysis of IGTE in resource-based cities of the Yellow River Basin

#### Temporal evolution characteristics

**Fig. 2:**
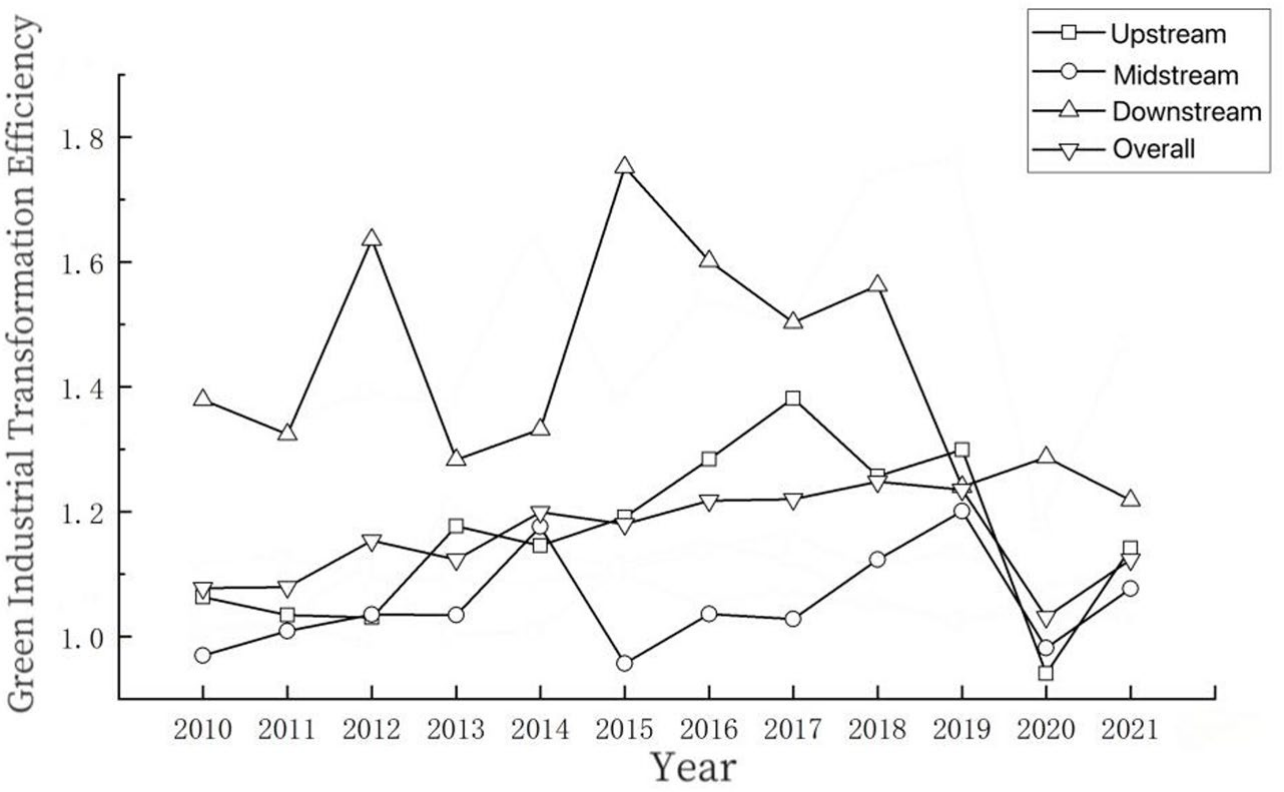
Trend of Industrial Green Transformation Efficiency in Resource-Based Cities by Geographic Location.

This study evaluates the IGTE of forty resource-based cities in the Yellow River Basin between 2010 and 2021 using MAXDEA 12.2 software. Figure 2 demonstrates the trend in average IGTE among resource-based cities in the Yellow River Basin and its headwaters, midstream, and downstream sections. According to the study, resource-based cities along the Yellow River experienced a significantly higher increase in the standard efficiency of IGTE between 2010 and 2021. Between 2010 and 2021, the average efficiency rose from 1.077 to 1.123. Notably, the efficiency increased significantly from 2015 to 2018, peaking at 1.248 in 2018. This surge was primarily driven by the implementation of China’s 13th Five-Year Plan in 2016. The plan explicitly called for “fostering new growth drivers in resource-depleted regions, industries in decline, and ecologically degraded areas; accelerating the development of successor industries; and promoting the innovative transformation of resource-based economies.” This policy significantly elevated the overall efficiency of IGTE across the Yellow River Basin.

The Yellow River meanders in a “几” form across nine provinces and autonomous areas. Because each province has a different level of economic growth and resource endowments, efficiency exhibits distinct regional variations. Comparing the basin, the lower reaches show the highest efficiency value, followed by the upper reaches, with the middle reaches ranking lowest. The IGTE in the lower reaches declined during the study period, dropping from 1.379 in 2010 to 1.219 in 2021. The primary reason for this decline in the lower reaches lies in the structural challenges faced by these regions: high volumes of industrial solid waste generation and storage, coupled with limited channels for comprehensive utilization. Furthermore, the dominance of heavy industry in their industrial structure makes it difficult to achieve solid waste reduction targets^56^. However, the overall efficiency level remains exceeding the levels observed in the middle and upper reaches. Leveraging their relatively developed economies, the lower reaches possess stronger technological capabilities and financial support, enabling them to drive industrial upgrading and green technological innovation. Concurrently, downstream regions actively implement national strategies. For instance, Shandong Province has adopted the “replacing old industries with new ones” strategy to drive the transformation of high-energy-consuming industries toward green and low-carbon development. The overall efficiency of IGTE in upstream regions saw a slight increase, rising from 1.063 in 2010 to 1.141 in 2021. Cities in the upstream region, such as Wuwei, Ordos and Zhangye, exhibit higher efficiency values. This is primarily because the upstream sections of the Yellow River exhibit relatively lower population density and heavy industrialization, resulting in reduced pressure on resource consumption and pollution emissions. Cities in the midstream region also show an upward trend in IGTE, rising from 0.969 in 2010 to 1.076 in 2021, though their overall level remains the lowest among the three regions. The middle reaches serve as a vital coal energy production base in China, characterized by substantial coal output, proven reserves, and development scale. This has fostered an energy industry system centered on coal mining, thermal power generation, and coal chemical processing. Prolonged dependence on a resource development model characterized by extensive practices, coupled with inefficient resource utilization and insufficient environmental governance investment, has resulted in persistently high industrial wastewater discharge intensity and severe water pollution exceeding standards. Consequently, the IGTE in the middle reaches of the Yellow River basin remains relatively low.

**Fig. 3:**
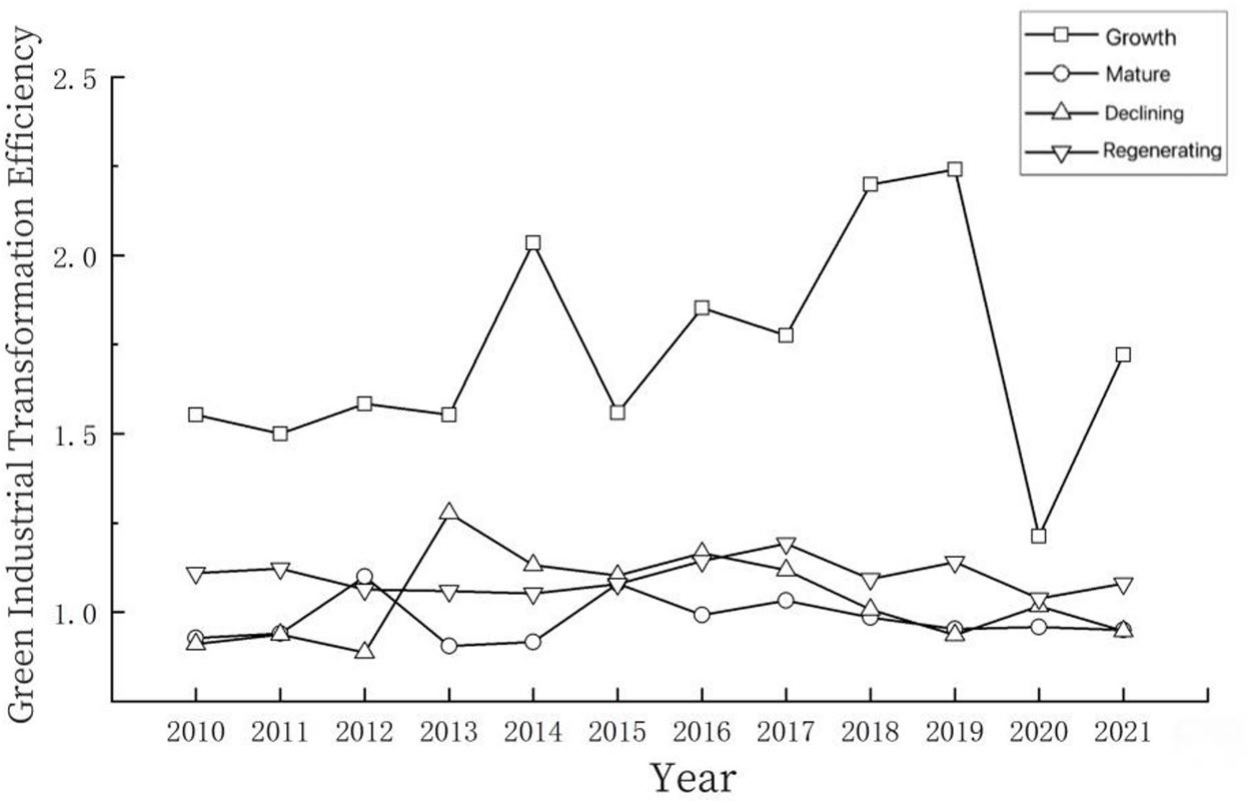
Trend of industrial green transformation efficiency in resource-based cities at different development stages.

Referring to the extensive definition of resource-based cities established as part of the Plan, the 40 resource-based cities examined in this study may be grouped into four types: expanding cities (8), mature cities (19), declining cities (7), and regenerating cities (6). Figure 3 illustrates the evolving trends in IGTE among these cities at different developmental stages within the Yellow River Basin. Overall, significant disparities exist in transition efficiency across city types: growing cities exhibit the highest industrial green efficiency, followed by regenerating cities, while declining and mature cities demonstrate relatively lower efficiency. The IGTE of growing cities increased substantially from 1.553 in 2010 to 1.721 in 2021. Growth-stage cities have lower development intensity and are in the early stages of resource development, minimal pollutant accumulation, and reduced environmental pressure. Their industrial sectors widely adopt green technologies and equipment featuring low energy consumption and emissions. Taking Longnan City as an example, while optimizing and expanding resource-dominated industries like nonferrous metallurgy and construction materials, the city actively adopted new technologies and equipment with green characteristics, such as low energy consumption and low emissions. This significantly improved resource utilization while reducing environmental pressure, resulting in high IGTE. Regenerative cities achieved an average IGTE of 1.098 between 2010 and 2021, with the fastest efficiency growth occurring from 2014 to 2017. Through the transformation of old and new growth drivers and the promotion of industrial upgrading, these cities gradually shifted from extensive resource utilization to green, low-carbon development. For instance, Zibo City implemented the “Environmental Protection Storm” campaign between 2013 and 2015, shutting down or relocating high-energy-consuming and high-polluting industries, thereby reducing industrial pollution emissions. Consequently, regenerative cities exhibit higher IGTE. Declining cities initially relied on high-pollution, high-energy-consumption industrial models. Upon entering the transformation phase, they faced constraints such as resource depletion, insufficient development momentum, and slow green technology upgrades, resulting in low IGTE. Mature cities saw their IGTE rise from 0.928 in 2010 to 0.949 in 2021, yet overall remained at a relatively low green transition level. Resource extraction in mature cities has stabilized, yet mining volumes remain high, and industrial structures exhibit significant rigidity. Take Changzhi City as an example: dominated by traditional heavy industries like coal, coking, and steel, it holds 12% of Shanxi Province’s proven coal reserves. While this resource-dependent model maintains short-term economic stability, it faces challenges, including industrial homogeneity and declining environmental carrying capacity. As the green and low-carbon transition advances, Changzhi urgently needs to optimize its energy structure, support the thoughtful restructuring of conventional industries, and foster emergent areas like high-end equipment manufacturing and new energy industries to reduce excessive reliance on coal resources.

#### Spatial distribution characteristics

**Fig. 4:**
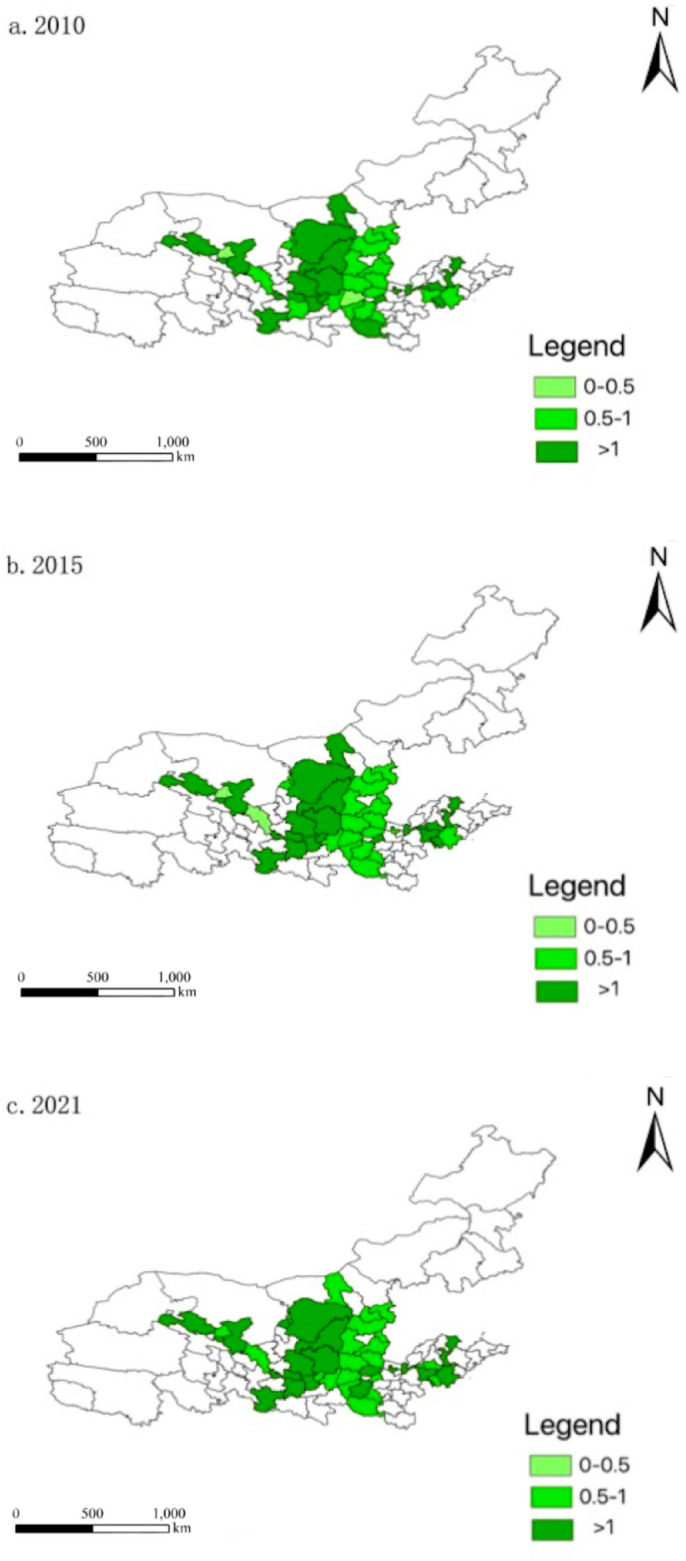
Spatial distribution of industrial green transformation efficiency.

This study utilized ArcGIS 10.8 software to visualize the IGTE of 40 resource-based cities in 2010, 2015, and 2021, with results presented in Fig. 4. Spatial evolution analysis reveals that efficiency values exhibited a “high at both ends, low in the middle” spatial distribution pattern throughout the study period. In 2010, efficiency in upstream and downstream regions significantly exceeded that of midstream areas. Cities such as Pingliang, Ordos, Wuwei, Hebi and Zaozhuang formed distinct clusters of high values, while resource-based cities in midstream regions like Weinan and Datong generally recorded lower efficiency scores. By 2015, the average IGTE had increased by 10% compared to 2010. Resource-based cities in downstream regions, such as Zibo, saw a 14% increase in IGTE. This was primarily driven by the 2011 Industrial Transformation and Upgrading Plan (2011–2015), which called for accelerating the elimination of outdated production capacity, vigorously promoting industrial energy conservation and consumption reduction, developing circular economies and remanufacturing industries, and enhancing green output levels. By 2021, regional disparities in IGTE among resource-based cities within the basin had gradually narrowed. The efficiency levels of Jinchang and Baiyin, two resource-based cities, rose from low to medium levels, while cities with low efficiency completely disappeared. Longnan, Dongying and Zhangye maintained relatively high levels of IGTE. In contrast, midstream cities continue to underperform, primarily due to the concentration of major coal resource-rich areas in this region, which has long led to a “high-carbon lock-in” effect. This effect has resulted in midstream cities maintaining a production structure dominated for an extended period by coal mining, coal chemical processing, and other primary processing industries. The industrial structure is relatively singular, characterized by high energy consumption and high emissions, while transformation efforts face significant sunk costs. In terms of technological pathways, these cities have long relied on traditional coal processing technologies, with insufficient investment in green technology research and development and weak technological reserves, making it difficult to overcome development bottlenecks. In terms of factor allocation, core factors such as capital and labor have long been concentrated in coal-related industries, leaving green industries inadequately supported, and issues of factor misallocation are particularly pronounced. This evolution demonstrates that IGTE exhibits not only significant spatial heterogeneity but also dynamic evolution influenced by a complex interplay of factors, including locational conditions, industrial foundations, and policy regulation.

### Analysis of drivers for IGTE in resource-based cities of the Yellow River Basin

#### Analysis of tobit model regression results

The current research leverages Stata 18.0 to conduct Tobit regression analysis on determinants impacting IGTE over the entire Yellow River Basin, its middle and upper reaches, lower reaches, and urban development stages. The regression results are presented in Tables 3 and 4.

(1) Economic Development Level (Lnpgdp): Economic development level demonstrates a considerable favorable impact on industrial environmental efficiency in resource-based cities, indicating that higher economic development levels can enhance IGTE. Cities with advanced economic development are better positioned to attract high-tech talent, providing the material foundation and technological support for urban industrial green development, thereby boosting IGTE. This finding aligns with the conclusions drawn from previous related studies and is consistent with the theoretical expectations of the Environmental Kuznets Curve (EKC), which posits that after economic development surpasses a certain stage, it provides robust support for improving environmental quality and advancing green transformation^57^.

(2) Technological Level (Lntec): The negative coefficient of technology level suggests that technological progress has not improved IGTE in resource-based cities. In these cities, green innovation is often embedded in sectors such as resource extraction and primary processing, while technological improvements enhance production capacity, they simultaneously stimulate scale expansion by reducing production costs. Consequently, total energy consumption and pollution emissions increase, partially offsetting the environmental benefits brought by technological innovation. Therefore, the observed negative relationship does not imply that green innovation itself is ineffective, it highlights the necessity for further improvements in the quality of green technologies and the application efficiency of green innovation in resource-based cities.

(3) Environmental Regulation (Er): At the 1% level, the predicted coefficient is notably positive, suggesting that increasing the intensity of environmental regulations increases the efficiency of the industrial green transition. Enhanced government environmental regulation compels industrial enterprises to adopt advanced, green, and efficient production processes. This not only increases industrial effective output but also reduces emissions of “three wastes” (wastewater, waste gas, and solid waste) during industrial production, thereby improving both environmental and economic performance^58^. This partially confirms the existence of the “Porter Hypothesis” in the Yellow River Basin, where current environmental regulations can enhance the efficiency of IGTE. This conclusion is consistent with Hao et al.^59^, supporting the view that appropriate environmental regulation can incentivize enterprises to pursue technological innovation and efficiency improvements.

(4) Industrial Structure (Is): The regression coefficient for industrial structure is negative but not statistically significant. This may stem from the dominance of heavy industry and manufacturing in most resource-based cities within the Yellow River Basin, coupled with the relatively sluggish development of emerging industries and modern services. This result differs from some studies that found industrial structure advancement significantly promotes green efficiency^60^. A potential reason lies in the study’s focus on resource-dependent cities located in the Yellow River Basin, which may not yet be effectively leveraging industrial structure optimization to drive green transformation. The tertiary sector in these cities might still be primarily composed of low-end services, with weak linkages and synergies with industry, failing to effectively absorb industrial overcapacity or provide green technological support.

(5) Trade Openness (Lntrade): The coefficient for trade openness is negative and passes the significance test, indicating that trade openness exerts a suppressing effect on IGTE. This result supports the applicability of the “pollution haven hypothesis” at the regional level: Given the relatively limited the growing economy of mineral-dependent communities in the Yellow River Basin, local governments may tend to lower environmental entry barriers during investment attraction. This leads to pollution-intensive industries relocating from regions with stricter environmental regulations to their areas, thereby hindering the green transition process. This finding is consistent with the conclusions reported in previous national-level studies^61^, highlighting the significant regional variation in the “double-edged sword” effect of trade openness. In the process of integrating into the global value chain, resource-dependent cities across the Yellow River Basin may have undertaken more low-end, high-pollution industrial segments, thereby exacerbating environmental pressure.

(6) Industrial Agglomeration (Lnind): Industrial agglomeration exerts a positive influence on IGTE, indicating that industrial clustering promotes economies of scale in industrial growth. This growth primarily reduces resource consumption and pollution intensity per unit output through economies of scale, while also facilitating the centralized supply and sharing of environmental technologies, facilities, and services, thereby enhancing IGTE^62^.This result aligns with findings from research in other industrial regions, confirming that moderate industrial agglomeration can improve green efficiency through sharing, matching, and learning effects.

Analyzing by mid-upstream and downstream sub-basins:

(1) Mid-upstream: Environmental regulations, industrial structure, trade openness, economic development level, and industrial agglomeration in resource-based cities of the mid-upstream region all exhibit consistent effects on IGTE with the basin as a whole. However, the inhibitory effect of technological level on IGTE in the mid-upstream region is not significant, likely because the relatively low technological level in the area constrains its influence on efficiency.

(2) Downstream: Environmental regulations, industrial structure, trade openness, and economic development levels in downstream cities positively influence IGTE, while technological level and industrial agglomeration exert a suppressing effect. This is consistent with Song et al. Findings^63^.Among these, the roles of environmental regulations and industrial structure are not significant. Downstream regions possess locational advantages, including numerous ports and convenient transportation. Strict environmental standards in developed countries exert a “backward pressure effect” on industrial enterprises in the lower reaches of the Yellow River through export trade, driving IGTE. Therefore, increased trade openness promotes the efficiency of IGTE in the downstream. In the industrial energy consumption structure of the lower Yellow River region, traditional energy sources like coal account for a high proportion, while the use of renewable and low-carbon energy sources remains relatively low. This energy structure leads to high carbon emissions and severe environmental pollution, exceeding the environment’s carrying capacity and resulting in reduced efficiency of IGTE. Therefore, industrial agglomeration has an unfortunate effect on the productivity of IGTE. In the downstream region, the crowding effect and the overloading of environmental carrying capacity caused by excessive agglomeration may have outweighed the positive effects of economies of scale. This is consistent with the structural predicament in the downstream region identified by Dong et al.^64^.

**Table 3:** Regression results of industrial green transformation efficiency. Values in parentheses represent t-statistics, * *p* < 0.1, ** *p* < 0.05, *** *p* < 0.01.

Variable	(1)	(2)	(3)
	Yellow River Basin	Upper-middle	Downstream
Lnpgdp	0.665*** (0.073)	0.429*** (0.076)	1.094*** (0.216)
Lntec	-0.146** (0.046)	-0.064 (0.048)	-0.564*** (0.156)
Er	0.639*** (0.074)	0.453*** (0.084)	0.175 (0.184)
Is	-0.040 (0.082)	-0.040 (0.080)	0.148 (0.388)
Lntrade	-0.088** (0.030)	-0.179*** (0.031)	0.404*** (0.111)
Lnind	0.107* (0.052)	0.120* (0.057)	-0.565*** (0.144)
cons	-5.622*** (0.870)	-2.221* (0.993)	-9.775*** (2.119)

Analyzing resource-based cities by development stage reveals that factors influencing IGTE vary across different stages.

1. For growth-stage cities, environmental regulations and economic development levels positively impact IGTE, while industrial agglomeration negatively affects it. This is consistent with Liu et al.^65^.Growth-stage cities are at a critical juncture of industrial upgrading. Stringent environmental regulations prompt industrial enterprises to adopt clean technologies, and higher green innovation is supported financially and commercially by economic development levels. Typically dominated by secondary industries during early industrialization, growth-stage cities rely primarily on local resource endowments like minerals. While industrial agglomeration fosters economies of scale, it also exacerbates resource consumption and pollutant emissions, thereby suppressing green transition efficiency.
2. For mature cities, IGTE is positively influenced by environmental regulations, industrial structure, economic development levels, and industrial agglomeration, while being negatively affected by technological advancement. Mature cities possess relatively well-developed industrial systems and policy environments. Stringent environmental regulations drive industrial restructuring and resource efficiency, while higher economic development provides the material foundation for technological advancement. This facilitates optimization of energy consumption structures and fuels IGTE. However, advanced technological capabilities may incentivize industrial enterprises to develop profit-driven products, neglecting environmental benefits and social sustainability, thereby suppressing green transformation efficiency.
3. For declining cities, industrial agglomeration positively impacts the efficiency of IGTE, while trade openness negatively affects it. For declining cities, the positive effect of industrial agglomeration primarily stems from the spatial clustering of replacement and alternative industries, sectors including renewable energy, energy saving, environmental protection, and advanced equipment manufacturing. This result echoes the findings of Wu et al. on the industrial transformation paths of resource-based cities in the Yellow River Basin, confirming the importance of cultivating replacement and alternative industries for declining cities^63^. Under the pressure of resource depletion, the agglomeration of these emerging industries facilitates the sharing of infrastructure, lowers innovation costs, and promotes green technology spillovers, thereby injecting new momentum into industrial green transformation. However, declining cities generally exhibit weak industrial competitiveness, and high trade openness may exacerbate two negative effects: first, path dependence on low-end, high-pollution industries; second, attracting the relocation of pollution-intensive industries. Both effects hinder the IGTE of declining cities in the Yellow River Basin.
4. The economic development level and industrial agglomeration of regenerating cities promote the efficiency of IGTE, while trade openness inhibits it. Regenerating cities in the early stages of transition may face increased international market competition pressure from heightened trade openness. Some industrial enterprises may lower green production standards to reduce costs, thereby inhibiting the efficiency of the IGTE.

**Table 4:** Regression results of industrial green transformation efficiency in resource-based cities at different stages of development. Values in parentheses represent t-statistics, * *p* < 0.1, ** *p* < 0.05, *** *p* < 0.01.

Variable	(1)	(2)	(3)	(4)
	Growing City	Mature City	Declining City	Regenerating City
Lnpgdp	0.707** (0.231)	1.169*** (0.130)	-0.106 (0.150)	0.196* (0.083)
Lntec	0.044 (0.124)	-0.490*** (0.073)	-0.001 (0.068)	0.048 (0.047)
er	0.615* (0.273)	0.481*** (0.100)	0.104 (0.131)	0.123 (0.076)
Is	-0.271 (0.172)	0.387** (0.144)	-0.019 (0.186)	0.036 (0.107)
Lntrade	-0.129 (0.116)	0.060 (0.041)	-0.129* (0.052)	-0.260*** (0.047)
Lnind	-0.427* (0.206)	0.358*** (0.067)	0.281*** (0.073)	0.242** (0.089)
cons	-2.725 (2.591)	-13.300*** (1.394)	2.014 (1.882)	0.507 (0.945)

#### Robustness tests

To verify the reliability of the empirical findings, this study recalculated the efficiency values for IGTE using the super-efficiency EBM model. These new efficiency values were then treated as the dependent variable in a panel Tobit regression, while keeping the explanatory variables unchanged. The findings of the Tobit model regression examination, utilizing the super-efficiency EBM estimates and relevant influencing factors, are presented in Table 5.

**Table 5:** Robustness Test 1. Values in parentheses represent t-statistics, * *p* < 0.1, ** *p* < 0.05, *** *p* < 0.01.

Variable	(1)	(2)	(3)	(4)
	Growing City	Mature City	Declining City	Regenerating City
Lnpgdp	-0.136*** (0.037)	0.139 (0.079)	-0.143* (0.056)	0.152*** (0.042)
Lntec	0.018 (0.020)	-0.085 (0.044)	0.068** (0.025)	-0.010 (0.023)
er	-0.076 (0.433)	0.152* (0.060)	-0.157 (0.484)	0.104** (0.031)
Is	-0.090** (0.027)	0.188* (0.087)	-0.044 (0.069)	-0.027 (0.054)
Lntrade	0.012 (0.018)	-0.013 (0.025)	-0.098*** (0.019)	-0.127*** (0.023)
Lnind	-0.092** (0.033)	0.089* (0.040)	0.184*** (0.027)	0.177*** (0.045)
cons	2.926*** (0.411)	-1.073 (0.841)	2.301** (0.698)	-0.259 (0.473)

To further validate the regression results, this study employs three methods: the substitution variable approach, the substitution estimation method, and adjusting the sample interval. The variable substitution method involves comprehensively assessing three metrics: industrial SO₂ emissions, discharge of industrial wastewater, and release of industrial soot (dust)—and constructing an indicator system using the entropy method to measure the comprehensive level of environmental regulation. To mitigate potential bias caused by logarithmic transformation of zero values, the proportion of R&D investment is used as a substitute for the technological level variable, while all other variables are held constant. This system replaces the environmental regulation variable and technological level variable in the original model, while other variables remain unchanged, enabling a new Tobit regression. The substitution estimation method employs OLS regression to fit the explanatory variables. The sample period adjustment accounts for potential policy changes affecting IGTE. This study selects the 18th CPC National Congress as the dividing point, conducting robustness tests over the 2013–2021 period. The regression results are presented in Table 6.

**Table 6:** Robustness Test 2. Values in parentheses represent t-statistics, * *p* < 0.1, ** *p* < 0.05, *** *p* < 0.01.

Variable	(1)	(2)	(3)	(4)
	Replacing environmental regulation	Replacing technological level	Replacing estimation methods	Adjusting the sample period
Lngdp	0.484*** (0.076)	0.522*** (0.065)	0.665*** (0.074)	0.734*** (0.098)
Lntec	-0.127* (0.052)	0.140** (0.067)	-0.146** (0.047)	-0.136* (0.058)
er	0.962** (0.334)	61.71*** (7.411)	0.639*** (0.075)	0.638*** (0.085)
Is	0.051 (0.086)	-0.135* (0.074)	-0.040 (0.082)	0.027 (0.093)
Lntrade	-0.072* (0.032)	-0.135*** (0.027)	-0.088** (0.030)	-0.108** (0.037)
Lnind	0.138* (0.058)	-0.005 (0.049)	0.107* (0.053)	0.106 (0.065)
cons	-4.308*** (0.935)	0.512*** (0.033)	-5.622*** (0.877)	-6.238*** (1.144)

## Conclusions

This study utilizes panel data from 40 resource-based cities in the Yellow River Basin between 2010 and 2021 as its sample, employing the Super-SBM model to measure IGTE. It compares and analyzes the spatiotemporal evolution characteristics of resource-based cities in the Yellow River Basin and further applies the Tobit model to examine the factors influencing IGTE across different developmental stages of these cities. Key findings are as follows:

(1) The IGTE of resource-based cities in the Yellow River Basin exhibited an overall fluctuating upward trend within the study period. Regional disparities in IGTE existed but showed a narrowing trend, with the lowest efficiency observed in the middle sections, and then in the upper sections, and the highest in the lower reaches. From the perspective of resource-based city development stages, the average IGTE ranking along the Yellow River basin is: growing cities > regenerating cities > declining cities > mature cities.

(2) With respect to the aggregate determinants affecting resource-dependent cities across the Yellow River Basin, environmental regulations and economic development levels significantly promote IGTE, while technological level and trade openness inhibit it. Regarding regional differences: In the middle and upper reaches, the impacts of environmental regulations, trade openness, and economic growth on IGTE are consistent with the patterns observed across the entire basin. - In the lower reaches, trade openness and economic development promote IGTE, while technological level and industrial agglomeration significantly inhibit it.

(3) Regarding the heterogeneity of influencing factors across urban development stages: For growing cities, IGTE is positively influenced by environmental regulations and economic development levels, while being negatively affected by industrial agglomeration. For mature cities, environmental regulations, industrial structure, economic development levels, and industrial agglomeration promote IGTE, whereas the technological level inhibits it. Industrial agglomeration enhances IGTE in declining and regenerating cities, while trade openness exhibits a significant inhibitory effect.

In light of these results, the following policy recommendations are offered:

First, given the generally low IGTE in resource-based cities along the Yellow River Basin, industrial transformation efforts should prioritize the application of green advanced technologies to enhance energy and resource productivity per unit output, thereby promoting pollution reduction, carbon emission cuts, quality improvement, and efficiency gains. Governments should strengthen environmental regulations, urging industrial enterprises to reduce pollution emissions of “three industrial wastes” through technological upgrades and enhance the comprehensive utilization of industrial resources. Emerging and future industries should be developed around new productive forces to drive traditional industry upgrades. Industrial enterprises should utilize the industrial internet for real-time energy consumption monitoring, forming new business models of “smart mining + green energy”.

Second, addressing regional disparities in IGTE of resource-based cities across upstream, midstream, and downstream sectors requires tailored approaches to promote regional coordination and green industrial development. Midstream and upstream regions can fully leverage abundant wind and solar resources in Ningxia and Inner Mongolia to deploy distributed renewable energy projects with supporting energy storage technologies and equipment. This provides stable, clean power for IGTE, achieving synergistic progress in ecological conservation and economic development. Downstream regions must optimize industrial structures by upgrading energy-intensive sectors like steel, chemicals, and building materials while providing financial support to guide enterprises toward high-end and low-carbon transformation.

Third, targeted policy recommendations should be formulated for resource-based cities at different development stages. Growing cities should prioritize controlling total resource exploitation and guiding industrial structural adjustment, strengthening environmental regulation and access standards avoiding lock-in effects from polluting industries. Extending resource-based value chains will gradually foster diversified development. Mature cities need to promote green technology application and industrial upgrading. Accelerating green technology diffusion improves resource efficiency, while better resource allocation enhances IGTE. Building high-quality green innovation systems will reinforce their leading role in industrial transformation. Declining cities should focus on fostering alternative industries, policy support is needed to stabilize employment and ease transitional pressures. Cultivating emerging industries and green services can create new growth drivers for long-term sustainability. Regenerating cities should develop successor and green industries while preventing pollution transfer. Building non-resource-dependent growth systems will ultimately lead to a diversified and green economic structure for long-term sustainability.

## Supplementary Information

Below is the link to the electronic supplementary material.

Supplementary Material 1

Supplementary Material 2

## Data Availability

Included in the Supplementary Information.The datasets used and analyzed during the current study are available from the corresponding author on reasonable request.
